# Causes and Risk Factors of Pediatric Spontaneous Intracranial Hemorrhage—A Systematic Review

**DOI:** 10.3390/diagnostics12061459

**Published:** 2022-06-13

**Authors:** Urszula Maria Ciochon, Julie Bolette Brix Bindslev, Christina Engel Hoei-Hansen, Thomas Clement Truelsen, Vibeke Andrée Larsen, Michael Bachmann Nielsen, Adam Espe Hansen

**Affiliations:** 1 Department of Diagnostic Radiology, Copenhagen University Hospital—Rigshospitalet, 2200 Copenhagen, Denmark; 2 Department of Pediatrics and Adolescent Medicine, Copenhagen University Hospital—Rigshospitalet, 2200 Copenhagen, Denmark; 3 Department of Clinical Medicine, University of Copenhagen, 2200 Copenhagen, Denmark; 4 Department of Neurology, Copenhagen University Hospital—Rigshospitalet, 2200 Copenhagen, Denmark

**Keywords:** pediatric spontaneous intracranial hemorrhage, pediatric hemorrhagic stroke, pediatric acute stroke

## Abstract

Previous studies suggest that the most common cause of spontaneous intracerebral hemorrhage in children and adolescents is arteriovenous malformations (AVMs). However, an update containing recently published data on pediatric spontaneous intracranial hemorrhages is lacking. The aim of this study is to systematically analyze the published data on the etiologies and risk factors of pediatric spontaneous intracranial hemorrhage. This systematic review was performed in compliance with Preferred Reporting Items for Systematic Reviews and Meta-Analyses (PRISMA) statement. A search in PubMed, Embase, Scopus, Web of Science and Cochrane Library was conducted aiming for articles published in year 2000 and later, containing data on etiology and risk factors of spontaneous intracranial hemorrhages in unselected cohorts of patients aged between 1 month and 18 years. As a result, forty studies were eligible for data extraction and final analysis. These included 7931 children and adolescents with 4009 reported etiologies and risk factors. A marked variety of reported etiologies and risk factors among studies was observed. Vascular etiologies were the most frequently reported cause of pediatric spontaneous intracranial hemorrhages (*n* = 1727, 43.08% of all identified etiologies or risk factors), with AVMs being the most common vascular cause (*n* = 1226, 70.99% of all vascular causes). Hematological and systemic causes, brain tumors, intracranial infections and cardiac causes were less commonly encountered risk factors and etiologies.

## 1. Introduction

Pediatric spontaneous intracranial hemorrhage is a rare but potentially disastrous emergency and often a diagnostic challenge. The young patients can have difficulties in communicating complaints, and the hemorrhage can be manifested by unspecific symptoms such as irritability, somnolence or headache, with lateralizing symptoms reported less frequently as compared to adult patients [1]. Unfortunately, this can lead to delayed stroke diagnosis in this patient group. The reported mortality after hemorrhagic stroke can be as high as one-third of the pediatric patients, and many develop long-term physical and psychosocial disability or seizures [2,3,4]. However, in contrast to adults, the pediatric brain may possess increased ability of tissue plasticity and repair after cerebrovascular lesions which may result in better outcomes after stroke in comparison to adults [5]. Although management principles can be similar to hemorrhagic strokes in adults, pediatric patients with spontaneous intracranial hemorrhage often possess distinct risk factors and etiologies.

Numerous conditions can lead to spontaneous intracranial hemorrhage in children, but due to rarity of this condition, large cohort analyses are scarcely reported. It is generally accepted that the most common etiology of spontaneous intracerebral hemorrhage in pediatric populations are arteriovenous malformations (AVMs), and the most common cause of spontaneous intracranial extracerebral hemorrhage in these populations is subarachnoid hemorrhage due to ruptured intracranial aneurysm [6]. The most recent systematic reviews on this subject find that AVMs remain the most common causes of pediatric intracerebral hemorrhage [7,8]. However, an update on this subject including both intra- and extracerebral hemorrhages and containing data from most recently published articles is lacking.

The aim of this systematic review is to provide an overview of the literature on pediatric spontaneous intracranial hemorrhage, with focus on etiologies and risk factors.

## 2. Materials and Methods

This systematic review was performed in accordance with Preferred Reporting Items for Systematic Reviews and Meta-Analyses (PRISMA) statement [9]. The search was conducted in PubMed, Embase, Scopus, Web of Science and Cochrane Library databases aiming for articles describing etiologies or risk factors of spontaneous intracranial hemorrhage in unselected cohorts of patients aged 1 month to 18 years. Intracranial hemorrhage in newborns below the age of 28 days is classified as perinatal stroke, a distinct entity with different risk factors from intracranial hemorrhages in infants and older children, and the age of 18 years is considered as the border between childhood and adulthood in most countries’ legal systems. The following types of intracranial hemorrhages were included: intracerebral, intraventricular, subarachnoidal, subdural and epidural hemorrhage. The search was performed on the 1 April 2022.

In our search strategy the following Medical Subject Headings (MeSH) terms were used: “Intracranial Hemorrhage*/etiology”[Majr:NoExp], “cerebral hemorrhage/etiology*”[Majr:NoExp], “stroke*/etiology*”[Majr:NoExp]), “Child”[MeSH Terms] and “Infant”[MeSH Terms], and keywords: “spontaneous intracranial haemorrhage”, “spontaneous intracranial hemorrhage”, “haemorrhagic stroke*”, “hemorrhagic stroke*”, “intracerebral haemorrhage*”, “intracerebral hemorrhage*”, “intra-cerebral haemorrhage*”, “intra-cerebral hemorrhage*”, “spontaneous cerebral hemorrhage”, “spontaneous cerebral haemorrhage”, “nontraumatic intracranial haemorrhage*”, “nontraumatic intracranial hemorrhage*”, “nontraumatic brain haemorrhage*”, “nontraumatic brain hemorrhage*”, “child*”, “childhood”, “infant*”, “risk factor*”, “etiology”, “aetiology”, “etiologic factors”, “aetiologic factors” and “cause*”. The asterisk (*) behind some keywords was used to find abstracts containing words starting with the same letters as the keyword.

Two independent authors (U.M.C. and J.B.B.B.) conducted the inclusion and exclusion of articles in the Cochrane technology platform, Covidence [10]. Inclusion criteria were retrospective or prospective cohort studies published in year 2000 and later providing etiologies or risk factors of pediatric spontaneous, non-traumatic intracranial hemorrhage in children and adolescents aged 1 month to 18 years. Exclusion criteria were articles describing etiologies in selected cohorts, i.e., hemorrhages only in children with known cancer, hemorrhages only in male population, etc., articles written in other language than English, case reports, reviews, conference abstracts and posters. Patients with hemorrhagic transformation of ischemic stroke were excluded from the analysis, as bleeding in the hemorrhagic transformation of ischemic infarction occurs secondary to ischemic stroke with its own risk factors. Traumatic intracranial hemorrhages were also excluded. However, articles with “minor head trauma” among other etiologies and risk factors were included [11,12,13], as minor falls are commonly encountered pediatric events, especially in younger children without well-developed gait and balance. Moreover, fall from standing height can also occur secondarily to spontaneous intracranial hemorrhage. Studies based on data from national registries were included in our analysis if other requirements were fulfilled. We considered “etiologies” as direct causes of pediatric spontaneous intracranial hemorrhage that could be documented on neuroimaging, such as hemorrhage caused by underlying AVM or brain tumor [14]. On the other hand, “risk factors” were considered as systemic vulnerability that results in increased risk of developing spontaneous intracranial hemorrhages, such as coagulator deficiency or sepsis.

Data quality in articles chosen after full text analysis was subsequently analyzed for bias in patient selection, flow and timing with Quality Assessment of Diagnostic Accuracy Studies 2 (QUADAS-2) method [15]. Disagreements after title and abstract screening were resolved by consensus between U.M.C. and J.B.B.B., and conflicts after full text review were resolved by consensus between all authors. The extracted data were etiologies and risk factors of spontaneous intracranial hemorrhage in the target patient group, the number of patients with each etiology or risk factor, the total number of patients analyzed in each article, types of intracranial hemorrhage analyzed, study type, patient age spectrum, and time period which patient data originate from.

## 3. Results

**Figure 1:**
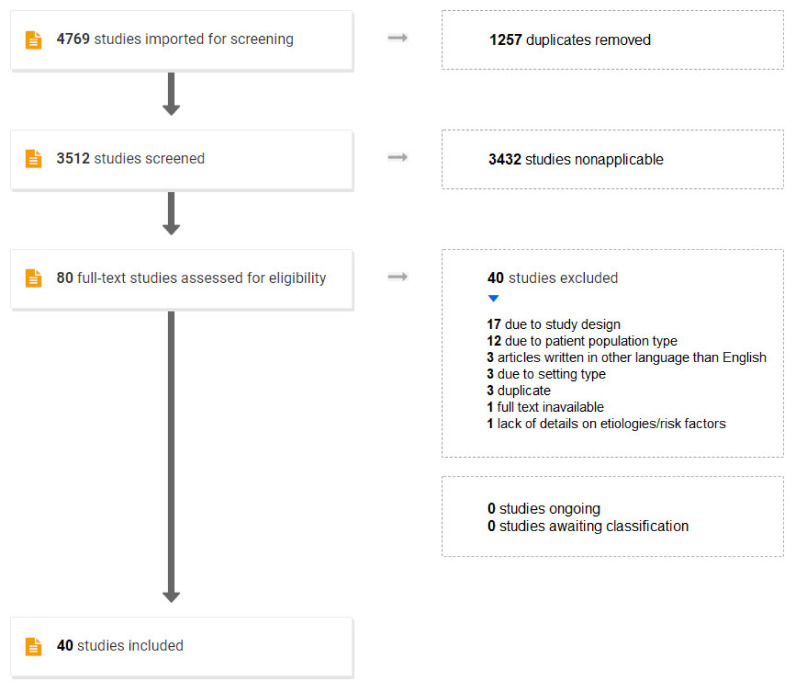
Pediatric spontaneous intracranial hemorrhage search strategy using PRISMA flowchart.

Our search yielded 3512 articles after removal of 1257 duplicates. The study selection process is summarized as the PRISMA flowchart (Figure 1). A total of 3432 studies were excluded as nonapplicable as they did not fulfill the inclusion criteria or described another subject than pediatric spontaneous intracranial hemorrhage. After full text review of 80 potentially relevant articles, 45 articles were included for the final data analysis with QUADAS-2. In the final data analysis, all studies except four [16,17,18,19] were rated as having low Risk of bias and low Concerns for applicability with respect to patient selection and having unclear Risk of bias and low Concerns for applicability with respect to Flow and Timing. The reason for unclear Risk of bias with respect to Flow and Timing was the potential risk of misdiagnosed hemorrhagic stroke in young children with unspecific symptoms and signs, which could result in omitting such patients in all articles. One article [20] was a duplicate. Data from the remaining 40 articles were extracted for the final data analysis [1,3,4,8,11,12,13,20,21,22,23,24,25,26,27,28,29,30,31,32,33,34,35,36,37,38,39,40,41,42,43,44,45,46,47,48,49,50,51,52] (see Table 1 and Table S1).

After data extraction from 40 articles, 7931 patients with 4009 diagnosed etiologies and risk factors were included in the final data analysis. Encountered etiologies and risk factors were summarized into seven groups: (**1**) **hematological causes** (consisting of etiologies or risk factors: “bleeding tendency”, “vitamin K deficiency”, “coagulator deficiency”, “hemophilia”, “thrombocytopenia”, “anemia” and “leukemia”), (**2**) **vascular causes** (encompassing “vascular abnormality”, “arterial malformation”, “arteriovenous malformation”, “cavernous aneurysm”, “Moyamoya disease”, “cavernoma”, “arteriovenous fistula” (AVF), “dural sinus malformation” (DSM), “vein of Galen malformation” (VGAM), “cerebral sinovenous thrombosis” (CSVT) and “vasculitis”), (**3**) **intracranial infection** (“intracranial infection” without further specification, “purulent meningitis”, “viral encephalitis”, “cerebral paragonimiasis” and “tuberculous infection”), (**4**) **brain tumor**, (**5**) **cardiac causes**, (**6**) **systemic causes** (consisting of other etiologies than hematological, vascular or cardiac) and (**7**) **unknown causes** (consisting of “undefined hemorrhagic stroke”, “unknown cause”, “cryptogenic hemorrhagic stroke”, “spontaneous intracranial hemorrhage” and “other etiology/condition” without closer details). In several studies the number of patients with diagnosed etiologies or risk factors was higher than the total number of analyzed patients, as some patients were known with more than one etiology or risk factor of spontaneous intracranial hemorrhage [3,11,12,29,34,35,40,51,52]. The frequency of each group of etiologies and risk factors was calculated as a percentage of the sum of all reported etiologies, risk factors and patients without known causes in each study.

An overview of the included studies with the proportion of etiologies and risk factors is presented in Table 1. In most reported patients, the cause or risk factor of spontaneous intracranial hemorrhage was unknown (*n* = 4135, 50.77%). This high number of cases with unknown etiology was driven to a large extent by one study; see below. Among established etiologies or risk factors, the most common were vascular causes (*n* = 1727, 43.08%), with AVMs being the most common undercategory (*n* = 1226, 70.99% of all vascular causes; Table 1 and Table S1). The 2nd most common etiology or risk factor were hematological causes (*n* = 1113, 27.76%), with coagulator deficiency as the most common hematological cause, (*n* = 345, 31.00% of all hematological causes, including 228 patients, 20.48% with induced coagulopathies such as vitamin K deficiency, use of anticoagulants, disseminated intravascular coagulopathy or liver failure). Systemic causes other than hematological, vascular or cardiac (*n* = 482, 12.02%), brain tumors (*n* = 376, 9.38%), intracranial infection (*n* = 157, 3.92%) and cardiac causes (*n* = 154, 3.84%) were less commonly reported. When excluding data from a single study where almost 75% of patients had an unknown etiology of spontaneous intracranial hemorrhage [35] (3295 patients out of 4424, 74.48%), vascular etiologies were still the most common cause (49.46% of patients with known etiologies and risk factors), followed by hematological causes (25.77%). Similar proportions still apply if studies with at least 30% of patients with unknown etiology or risk factor of pediatric spontaneous intracranial hemorrhage are excluded [11,13,35,36,39,42,44,47].

**Table 1:** Number of patients with different etiology- and risk factor categories in each of the included studies. Study type, time span, age of study participants and types of intracranial hemorrhages analyzed in each of the included articles are also provided. The most common and the second most common causes of spontaneous intracranial hemorrhage in each study are marked with brown and yellow colors, respectively. Abbreviations: N/A—not applicable, ICH—intracerebral hemorrhage, IVH—intraventricular hemorrhage, SAH—subarachnoidal hemorrhage, SDH—subdural hemorrhage, ITH—infratentorial hemorrhage, EDH—epidural hemorrhage, y—year, m—month, d—day.

Publication	Study Type *	Time Period	Number of Patients/Causes **	Age	Hemato-Logical Cause	Vas-Cular Cause	Intra-cranial Infection	Brain Tumor	Cardiac Cause	Systemic Cause ***	Unknown Cause	Comments—Hemorrhage Type Analyzed
Lanthier et al., 2000 [21]	Single/retrosp.	1991–1997	21	1 m–18 y	9.52%	66.67%	N/A	9.52%	N/A	N/A	14.28%	ICH with or without SDH, EDH or SAH.
Al-Jarallah et al., 2000 [3]	Single/retrosp.	1990–1998	68/79	3 m–18 y	26.58%	40.51%	1.26%	11.39%	N/A	2.53%	17.72%	ICH with or without SDH or SAH.
Sandberg et al., 2001 [22]	Single/retrosp.	1960–2000	3	43 d–3 m	66.67%	33.33%	N/A	N/A	N/A	N/A	N/A	ICH with SDH and IVH, ICH with SDH, ICH.
Suh et al., 2001 [23]	Single/retrosp.	1985–?	16	2 m–2 y	N/A	100.0%	N/A	N/A	N/A	N/A	N/A	ICH, SAH, SDH, IVH.
Blom et al., 2003 [4]	Single/retrosp.	1978–1998	56	1 m–16 y	17.86%	53.57%	5.36%	1.78%	N/A	1.78%	19.64%	ICH, IVH, SAH.
Meyer-Heim et al., 2003 [24]	Single/retrosp.	1990–2000	34	2 m–17 y	11.76%	73.53%	N/A	2.94%	N/A	N/A	11.76%	ICH, SAH, IVH, ITH, SDH.
Zahuranec et al., 2005 [25]	One county/retrosp.	2002–2003	5	2 m–17 y	N/A	40.00%	N/A	N/A	20.00%	20.00%	20.00%	ICH.
Aydinli et al., 2006 [26]	Single/retrosp.	1995–2003	22	40 d–8 y	77.27%	N/A	N/A	N/A	N/A	N/A	22.73%	Not specified, probably ICH.
Liu et al., 2006 [27]	Single/retrosp.	1997–2003	50	1 m–16 y	N/A	62.00%	N/A	10.00%	N/A	16.00%	12.00%	ICH with or without IVH or SAH.
de Ribaupierre et al., 2008 [20]	Dual/retrosp.	1995–2005	22	2 m–18 y	N/A	81.82%	N/A	N/A	N/A	N/A	18.18%	ICH, SAH.
Kumar et al., 2009 [28]	Single/retrosp.	1998–2007	50	2 m–17 y	4.00%	88.00%	N/A	4.00%	N/A	N/A	4.00%	ICH, SAH, IVH, intracerebellar hemorrhage.
Wang et al., 2009 [12]	Single/retrosp.	1996–2006	94/181	1 m–16 y	82.32%	7.73%	2.21%	N/A	N/A	0.55%	7.18%	SAH, ICH, other intracerebral hemorrhages.
Del Balzo et al., 2009 [29]	Triple/retrosp.	2001–2006	4/5	2 y–14 y	N/A	80.00%	N/A	N/A	N/A	20.00%	N/A	Hemorrhagic stroke, no further details provided.
Tham et al., 2009 [30]	Single/retrosp.	1999–2006	11	3 m–18 y	27.27%	36.36%	N/A	N/A	9.09%	N/A	27.27%	SAH, SDH, ICH, IVH.
Laugesaar et al., 2010 [31]	Regional + national registry/retrosp. + prosp.	1995–2006 (retrosp.), 2004–2006 (prosp.)	16	30 d–18 y	N/A	62.5%	6.25%	N/A	N/A	12.5%	18.75%	ICH, SAH.
Beslow et al., 2010 [32]	Single/prosp.	2006–2008	22	4 y–17 y	N/A	90.91%	N/A	N/A	N/A	N/A	9.09%	ICH, wthout or with IVH or other hemorrhage types.
Paonessa et al., 2010 [33]	Single/retrosp.	10 years (unspecified)	17	5 y–16 y	N/A	82.35%	N/A	N/A	N/A	N/A	17.65%	ICH, SAH.
Christerson et al., 2010 [34]	Regional registry/retrosp.	2000–2006	21/22	9 y–16 y	13.64%	77.27%	N/A	N/A	N/A	4.54%	4.54%	ICH, SAH.
Statler et al., 2010 [35]	Multistate registry/retrosp.	2000–2003	4424/4425 ****	1 m–18 y	8.38%	6.85%	2.24%	6.15%	1.74%	0.18%	74.46%	Hemorrhagic stroke, no further details provided.
Yock-Corrales et al., 2011 [36]	Single/retrosp.	2003–2008	31	1 m–16 y	N/A	61.29%	N/A	N/A	N/A	N/A	38.71%	ICH and “unspecified intracranial hemorrhage” (ICD-10 codes).
Zidan et al., 2012 [37]	Single/retrosp.	2008–2009	17	1 m–18 y	52.94%	35.29%	N/A	11.76%	N/A	N/A	N/A	ICH.
Lo et al., 2013 [38]	Single/retrosp.	2000–2009	59	0.1 y–18 y	3.39%	52.54%	1.69%	18.64%	8.47%	8.47%	6.79%	SAH with ICH or IVH, ICH, IVH with other hemorrhage types.
Kalita et al., 2013 [39]	Single/retrosp.	2001–2011	10	1 m–18 y	N/A	40.00%	N/A	N/A	N/A	20.00%	40.00%	ICH only.
Beslow et al., 2013 [40]	Triple/prosp.	2007–2012	53/54	28 d–18 y	20.37%	62.96%	N/A	N/A	N/A	N/A	16.67%	ICH, IVH, ICH with IVH.
Xie et al., 2014 [11]	Single/retrosp.	2003–2011	109/201	1 m–18 y	67.16%	8.95%	5.97%	0.99%	0.50%	N/A	16.42%	SAH, ICH, SDH, other non-traumatic hemorrhage.
Deng et al., 2015 [41]	Single/retrosp.	2002–2011	249	1 m–18 y	N/A	45.78%	N/A	N/A	N/A	37.35%	16.87%	ICH, SAH.
Adil et al., 2015 [42]	Multistate registry/retrosp.	2003, 2006, 2009	1172	1 y–18 y	15.95%	17.58%	N/A	N/A	1.88%	24.57%	40.02%	ICH and ICH with SAH.
Liu et al., 2015 [43]	Single/prosp.	2012–2014	70	1 y–18 y	1.43%	74.28%	N/A	2.86%	N/A	1.43%	20.00%	ICH with or without IVH or SAH.
Gelfand et al., 2015 [44]	State registry/retrosp.	1997–2007	42	2 y–17 y	N/A	66.67%	N/A	N/A	N/A	N/A	33.33%	Hemorrhagic stroke, no further details provided.
Abbas et al., 2016 [45]	Single/retrosp.	2007–2014	50	1 m–16 y	56.00%	14.00%	4.00%	N/A	N/A	N/A	26.00%	ICH, SAH, SDH, IVH.
Chiang et al., 2018 [46]	National registry/retrosp.	2010–2011	299	1 m–18 y	9.70%	30.77%	9.36%	11.37%	8.70%	17.39%	12.71%	Hemorrhagic stroke, no further details provided.
Yock-Corrales et al., 2018 [13]	Single/retrosp.	7 y, unspecified.	34	1 m–18 y	14.70%	35.29%	N/A	N/A	N/A	N/A	50.00%	5 patients with SAH, no details on the remaining.
Uzunhan et al., 2019 [47]	Single/retrosp.	2007–2013	12	1.5 y–13 y	16.67%	41.67%	N/A	8.33%	N/A	N/A	33.33%	ICH or SAH.
Söbü et al., 2019 [48]	Single/retrosp.	2000–2011	15	1 m–18 y	73.33%	6.67%	6.67%	N/A	N/A	6.67%	6.67%	ICH, SAH.
de Montferrand et al., 2019 [49]	Single/retrosp.	1992–2015	105	1 m–15 y	1.90%	88.57%	N/A	N/A	N/A	N/A	9.52%	Hemorrhagic stroke, no further details provided.
Gerstl et al., 2019 [1]	Single/retrosp.	2010–2016	33	1 m–17 y	6.06%	39.39%	3.03%	42.42%	N/A	6.06%	3.03%	ICH, IVH, SAH.
Boulouis et al., 2021 [8]	Single/retrosp. + prosp. *****	2000–2019	243	28 d–18 y	5.76%	77.37%	N/A	2.88%	6.99%	0.82%	6.17%	ICH with or without IVH.
Huang et al., 2021 [50]	Dual/retrosp.	2008–2020	200	29 d–18 y	33.50%	37.00%	1.50%	3.50%	N/A	2.50%	22.00%	ICH with or without IVH or SAH.
Deng et al., 2021 [51]	Multi/retrosp.	2018–2018	140/152	1 m–18 y	N/A	78.95%	N/A	2.63%	1.97%	3.29%	13.16%	ICH and SAH.
Pangprasertkul et al., 2022 [52]	Single/retrosp.	2009–2018	32/39	1 m–18 y	61.54%	28.21%	2.56%	N/A	2.56%	2.56%	2.56%	Radiological evidence of intracranial hemorrhage.

* The following study types were encountered: single-, dual-, triple- and multiple-center studies, and studies based on regional/state/national registries. Retrosp.—retrospective, prosp.—prospective. ** In some studies, patients had more than one etiology or risk factor of spontaneous intracranial hemorrhage. In these cases, the number of analyzed patients is written first, followed by the sum of all diagnosed etiologies, risk factors and the number of patients with unknown cause of spontaneous intracranial hemorrhage. The frequency of each group of etiologies and risk factors was calculated as a percentage of this sum. *** Other than hematological, vascular or cardiac causes. **** Data calculated from a percentage bar graph in the article, a minimal measurement error is possible. ***** Based also on a prospective hospital registry.

As visualized in Table 1, in 29 of the 40 studies analyzed, the most common reported cause was vascular [3,4,8,13,20,21,23,24,25,27,28,29,30,31,32,33,34,36,38,39,40,41,43,44,46,47,49,50,51]; hematological disorders were the most common cause in nine studies [11,12,22,26,35,37,45,48,52]; brain tumor was encountered in most patients from one study [1], and systemic causes were most frequent in one study [42].

Marked differences between some studies are notable regarding the number of patients, occurrence of different categories and subcategories of etiologies and risk factors and the number of patients without known etiology or risk factors of spontaneous intracranial hemorrhage among analyzed subjects (Table 1 and Table S1). Most studies were single-center (28 out of 40) and retrospective (37 out of 40, including two studies [8,31] that investigated both prospective and retrospective data).

## 4. Discussion

This systematic review with analysis of data published in the last twenty-two years confirms that vascular causes remain the most common etiologies or risk factors of spontaneous intracranial hemorrhages in children and adolescents aged between 1 month and 18 years. Among vascular causes, AVMs remain the most common cause of pediatric spontaneous intracranial hemorrhage, with other vascular subcategories being rare. This review also reveals that hematological causes are the second most common cause, with coagulopathy being the most frequent cause in this group. Overall, a considerable variability of reported etiologies and risk factors of pediatric spontaneous intracranial hemorrhage is characteristic among the included studies. Referral bias can be the possible explanation, as many studies are based on internal patient registries from specialized, tertiary care departments (neurosurgical, hematological, etc.) and do not necessarily represent the general population or include patients after extensive diagnostic work-up.

Most of the studies involved patients with intracerebral hemorrhage with or without intraventricular extension, many studies examined subarachnoidal hemorrhages, while subdural and epidural hemorrhages were infrequently reported (Table 1). Imaging appearance of hemorrhage in different intracranial compartments is described elsewhere [14]. Analysis of most common etiologies and risk factors per hemorrhage in different intracranial compartments could not be performed, as the vast majority of studies do not provide such data. The reason for exclusion of subdural and epidural hemorrhages from most studies of pediatric spontaneous intracranial hemorrhages could be the frequent connection between these two types of intracranial hemorrhage and trauma. However, especially in the youngest children learning to walk and developing the sense of balance, exclusion of minor trauma preceding the intracranial hemorrhage, such as falls from standing height, can be difficult. Therefore, we included articles reporting minor trauma among other etiologies and risk factors if the number of patients with minor trauma could not be subtracted from the total number of patients [11,12,13]. We have also included one study reporting patients with major trauma among other causes of spontaneous intracranial hemorrhage [46] and another study which included few patients with “brain trauma” among causes of hemorrhagic stroke [51] as the number of such patients was small in both articles, and it was not possible to subtract the number of these patients from the total. As the majority of articles did not provide causes of pediatric spontaneous intracranial hemorrhage according to the age of particular patients, age subgroup analysis could not be performed.

In some of the included studies, there was a high number of patients with unknown cause of intracranial hemorrhage, exceeding 50% [35] or 30% of reported patients [11,13,35,36,39,42,44,47]. This may be explained by data sources from large national or multistate patient registries [35,42] with different requirements for data registration depending on the aim of the registry. Some national registries are known to contain information only of particular interest, as in the article by Tuppin et al. [17] where only data relevant for insurance issues were collected. Data search and analysis in the included registry-based studies were primarily based on ICD-9-CM codes (The International Classification of Diseases, 9th revision, Clinical Modification, [53]) [35,42,46], without providing details on the use of neuroimaging during workup of the included patients. However, the use of neuroimaging for confirmation of the intracranial hemorrhage has to be expected in these studies analyzing data from year 2000 and onwards (Table 1). Still, after exclusion of the article by Statler et al. containing ¾ of patients with unknown cause of hemorrhagic stroke [35], the proportion of etiologies and risk factors remained roughly the same, with vascular and hematological factors being the two most common causes. Smaller, single-center studies with lower number of patients typically had lower percentage of patients labelled as with unknown cause of intracranial hemorrhage, perhaps due to more extensive testing for possible etiologies.

A marked variety of etiologies and risk factors of pediatric spontaneous intracranial hemorrhage among the analyzed studies can be observed (Table S1). This concerns especially the systemic risk factors other than hematological, vascular or cardiac. Numerous etiologies and risk factors of pediatric spontaneous intracerebral hemorrhage are well-known and consistent with etiologies and risk factors of spontaneous intracranial hemorrhage in the adult population, as intracranial aneurysms, intratumoral bleeding or cerebral aspergillosis [54,55,56,57,58]. On the other hand, some etiologies and risk factors are more common in the pediatric population, such as inherited hematological causes or AVMs [54].

A recently published systematic review on etiologies and risk factors of pediatric intracerebral hemorrhage by Boulouis et al. [8] reported an aggregate prevalence of 0.59 of vascular lesions, 68.3% of which were AVMs, followed by hematological disorders with an aggregate prevalence of 0.12. In contrast to our study, Boulouis et al. studied only intracerebral hemorrhage without other types of intracranial hemorrhage, included articles older than the present review (starting from year 1990), where access to imaging with magnetic resonance imaging (MRI) may have been more restricted, and only included articles until 2019. Another recent systematic review by Gumer et al. [7] analyzed hemorrhagic stroke in subjects aged between 28 days and 20 years in studies published between the years 1997 and 2011, and concluded that vascular factors were the most common cause of pediatric hemorrhagic stroke (54%), with AVMs representing the most common subcategory of vascular causes (30%). Among the established etiologies and risk factors of pediatric hemorrhagic stroke, medical causes were the second most common causative factor of hemorrhagic stroke (9%). No further details on the specific medical causes were provided.

Our study has several limitations. Referral bias can be presumed, as especially the youngest children with unexpected, spontaneous intracranial hemorrhage can present to the emergency departments with unspecific symptoms and without lateralizing neurological signs, with potential risk of being misdiagnosed and excluded from further investigations. The accessibility to health services can also be limited in some parts of the world. Therefore, there is a possibility that not all children and adolescents with spontaneous intracranial hemorrhage may figure in hospital records, and our analysis of their etiologies and risk factors may not necessarily reflect the general population. Intracranial hemorrhages other than intracerebral were underreported in most of the included studies. Patients with underlying brain malignancy were excluded in some of the studies, and their number is therefore underestimated in our analysis. Most of the studies did not provide complete data records for each of the analyzed patients, and therefore, subgroup analyses, i.e., age subgroup analysis, could not be performed.

## 5. Conclusions

Vascular etiologies remain the most common cause of pediatric spontaneous intracranial hemorrhages, with AVMs being the most frequently reported subcategory. The second most common cause is hematological disorders, with coagulator deficiency being the most common risk factor in this group. Systemic causes, brain malignancies, intracranial infections and cardiac causes are less frequently reported etiologies and risk factors. A considerable variety of reported causes among studies was evident, as was the proportion of patients with an unknown cause of spontaneous hemorrhage between studies.

## Data Availability

Data are available from the cited studies that were included in this systematic review.

## Supplementary Materials

The following supporting information can be downloaded at: https://www.mdpi.com/article/10.3390/diagnostics12061459/s1.
